# Panduratin A, a Possible Inhibitor in Metastasized A549 Cells through Inhibition of NF-Kappa B Translocation and Chemoinvasion 

**DOI:** 10.3390/molecules18088764

**Published:** 2013-07-24

**Authors:** Shiau-Chuen Cheah, Siew-Li Lai, Sui-Ting Lee, A. Hamid A. Hadi, Mohd. Rais Mustafa

**Affiliations:** 1 Faculty of Medicine and Health Sciences, UCSI University, Kuala Lumpur 56000, Malaysia; 2 Department of Pharmacology, Faculty of Medicine, University of Malaya, Kuala Lumpur 50603, Malaysia; E-Mails: siewli_520@yahoo.com (S.-L.L.); suiting87@siswa.um.edu.my (S.-T.L.); rais@um.edu.my (M.R.M.); 3 Centre of Natural Products and Drug Discovery (CENAR), Department of Chemistry, Faculty of Science, University of Malaya, Kuala Lumpur 50603, Malaysia; E-Mail: ahamid@um.edu.my

**Keywords:** panduratin A, NF-κB, caspase, MMP-2, p53, p21

## Abstract

In the present study, we investigated the effects of panduratin A (PA), isolated from *Boesenbergia rotunda*, on apoptosis and chemoinvasion in A549 human non-small cell lung cancer cells. Activation of the executioner procaspase-3 by PA was found to be dose-dependent. Caspase-3 activity was significantly elevated at the 5 µg/mL level of PA treatment and progressed to a maximal level. However, no significant elevated level was detected on procaspase-8. These findings suggest that PA activated caspase-3 but not caspase-8. Numerous nuclei of PA treated A549 cells stained brightly by anti-cleaved PARP antibody through High Content Screening. This result further confirmed that PA induced apoptotic cell death was mediated through activation of caspase-3 and eventually led to PARP cleavage. Treatment of A549 cells with PA resulted in a strong inhibition of NF-κB activation, which was consistent with a decrease in nuclear levels of NF-κB/p65 and NF-κB/p50 and the elevation of p53 and p21. Besides that, we also showed that PA significantly inhibited the invasion of A549 cells in a dose-dependent manner through reducing the secretion of MMP-2 of A549 cells gelatin zymography assay. Our findings not only provide the effects of PA, but may also be important in the design of therapeutic protocols that involve targeting of either p53 or NF-κB.

## 1. Introduction

Currently, much attention is being focused on natural product-based therapeutics, especially phytochemicals, owing to numerous reports that have revealed the interference of phytochemicals in cancer-related pathways, thus conferring pharmaceuticals value beyond their traditional use [1,2]. Panduratin A (PA, CAS Registry Number: 89837-52-5; molecular weight for C_26_H_30_O_4_: 406.52), a natural chalcone from *Boesenbergia rotunda*, has been reported to exhibit anti-oxidant, anti-inflammatory and anti-cancer properties [3,4,5,6]. Recently, we isolated PA from the methanolic extract of *B. rotunda* [7] and in our previous study, we demonstrated antiproliferative and proapoptotic effect of this compound in human A549 non-small cell lung cancer cells and delineated the mechanism of this effect [1].

The tumour suppressor p53 inhibits cell growth through activation of cell-cycle arrest and apoptosis [8,9], and most cancers have either mutation within the p53 gene or defects in the ability to induce p53. Activation of p53 induces apoptosis in many tumour cells and may provide effective cancer therapy [10,11]. At the same time, p53 is one of the key proteins that modulates the apoptotic response is NF-κB, a transcription factor that can protect or contribute to apoptosis [12]. NF-κB is a ubiquitous transcription factor which plays an important role in many physiological processes, such as cell proliferation, cell death, inflammation and immune response [13,14]. Under resting conditions, NF-κB is present as an inactive heterotrimer which consists of p50, p65, and I kappa B alpha (IκBα) subunits in the cytoplasm. Following activation by numerous of stimuli, IκBα protein undergoes phosphorylation and degradation. Unbound p50–p65 heterodimer translocates to the nucleus, subsequently binds with specific DNA motif in the promoter regions of target genes and activates their transcription. Dysregulation of NF-κB is implicated in many types of human cancers [15,16].

p21 is often overexpressed in aggressive tumours, including carcinomas of the pancreas On the other hand, p21 is a member of the Cip/Kip family and identified as a cell cycle regulator or inhibitor through inhibition of different cyclin/cyclin-dependent kinase complexes [17,18,19,20]. In addition to its role in cell cycle control, p21 is involved in the regulation of gene transcription, apoptosis and is a downstream target of the tumour suppressor, breast, prostate, ovary and cervix [21,22]. MMPs are known for their ability to cleave several extracellular matrix constituents as well as non-matrix proteins [23]. Increased expression of MMPs was observed in several human diseases such as epithelial tumours [24] and cancer [25], suggesting an implication of these enzymes in the immune defence, inflammation, and repair mechanisms [26]. In particular, MMP-2, MPases is able to regulate the inflammatory process by cytokine and chemokine activation/inactivation [26,27]. Together these observations suggest that the role played by p21 and MMP-2 are important in inhibition of cancer cells. Therefore, targeting on the signaling pathway mentioned above could be able to halt tumor development. In this study, we will be focused on caspases, NF-κB/p65 and NF-κB/p50, p53, p51 and MMP-2.

## 2. Results and Discussion

Our previous study indicated that PA exhibited cytotoxicity, with an IC_50_ value of 4.4 μg/mL (10.8 μM) [1]. Referring to Lai *et al.* [28], PA was tested against WI-38 human fibroblast cells and WRL-68 human hepatic epithelial cells with IC_50_ values of 18.86 and 12.34 μM, respectively, at 24 h post-treatment using an MTT assay. On the hand, there was evidence that PA treatment had no to little effect on normal human epithelial and fibroblast cells [9], hence it’s suggested that PA has selective cytotoxicity towards cancer cells. PA arrested cancer cells labeled with bromodeoxyuridine (BrdU) and phosphohistone H3 in the mitotic phase. The cytotoxic effects of PA were found to be accompanied by a dose-dependent induction of apoptosis, as assessed by DNA condensation, nuclear morphology and intensity, cell permeability, mitochondrial mass/potential, F-actin and cytochrome c. In addition, treatment with an apoptosis-inducing concentration of PA resulted in significant inhibition of NF-κB translocation from cytoplasm to nuclei activated by TNF-α [1].

**Figure 1:**
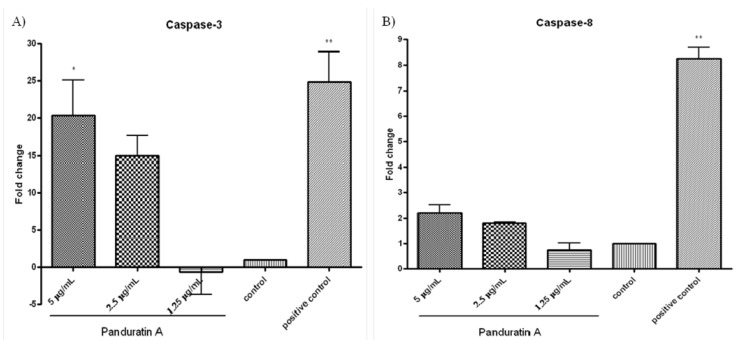
Effect of pandurartin A on caspases activation. Fold increase of the levels of (**A**) caspase-3 and (**B**) caspase-8 in A549 cells treated with various concentrations of PA, compared to vehicle control. The fluorescence intensity was measured at excitation wavelength of 390 nm and emission wavelength of 500 nm. The increase of protease activities was determined by comparing the levels in PA-treated A549 cells with the vehicle control.

Caspases are present in the proforms (inactive) and become active after site-specific cleavage to participate in the process of apoptosis. To determine whether caspases are involved in apoptosis induction by PA, the protein levels of active caspases in PA-treated cells were evaluated. Activation of the executioner procaspase-3 by PA was found to be dose-dependent (Figure 1A). Caspase-3 activity was significantly elevated at the 5 µg/mL of PA treatment and progressed to a maximal level (20-folds over vehicle control) after 24 h of incubation (Figure 1A). No significant elevated level was detected on pro-caspase-8 after the addition of 5 µg/mL PA over 24 h of incubation (Figure 1B). These findings suggest that PA activated caspase-3, but not caspase-8. 

**Figure 2:**
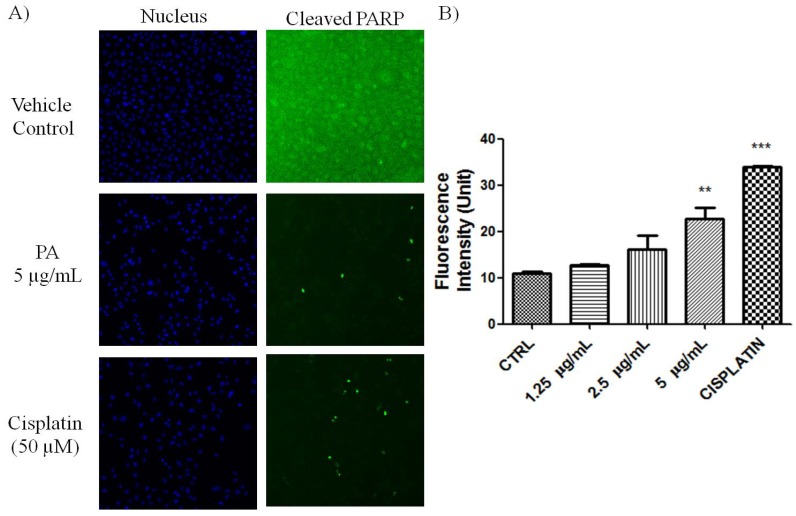
Induction of PARP cleavage in PA treated A549 cells. (**A**) Fluorescent images of A549 cells, treated with PA and cisplatin (positive control) for 24 h. Images were acquired using Cellomics ArrayScan reader (10× magnifications); (**B**) The fluorescence intensity was quantified by compartmental analysis Bioapplication. PA dose-dependently induced PARP-cleavage, as indicated by increased in fluorescence intensity in the nucleus.

**Figure 3:**
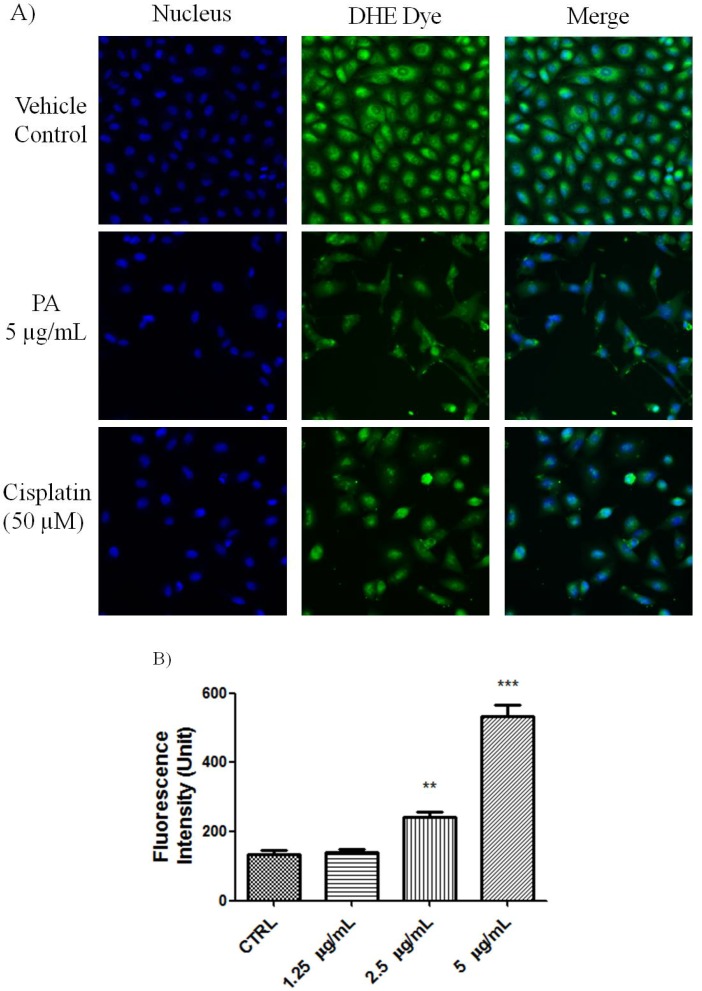
PA induced oxidative stress on A549 cells. (**A**) Fluorescent images of PA treated A549 cells stained with DHE dye. Cisplatin was included as positive control. PA induced oxidative stress was evidenced with increased ethidium dye, a product of DHE oxidation in the nuclues. Images were acquired using Cellomics ArrayScan reader; (**B**) The amount of ethidium, was quantified by compartmental analysis Bioapplication.

PARP cleavage is an essential marker for caspase 3-mediated apoptosis. PA treated A549 cells showed positive in the HCS staining using antibody that is specific for cleaved poly (ADP-ribose) polymerase (PARP). As shown in Figure 2, numerous nuclei of PA treated A549 cells stained brightly by anti-cleaved PARP antibody. As positive control, Cisplatin also induced PARP cleavge in A549 cells. This result further confirmed that PA induced apoptotic cell death was mediated through activation of caspase-3 and eventually led to PARP cleavage. Reactive oxygen species such as superoxide and hydrogen peroxide can influence p53-mediated and TNF-α triggered cell death. We next investigated if the NF-κB inhibition as well as p53 activation of PA could be mediated by ROS. As showed in Figure 3, PA treatment resulted in increased in oxidative stress in a dose-dependent manner (1.25–5 μg/mL). PA induced increase in ROS, oxidised the DHE dye into ethidium, which bound to the DNA. Cisplatin was included as positive control, also induced increased in oxidative stress.

**Figure 4:**
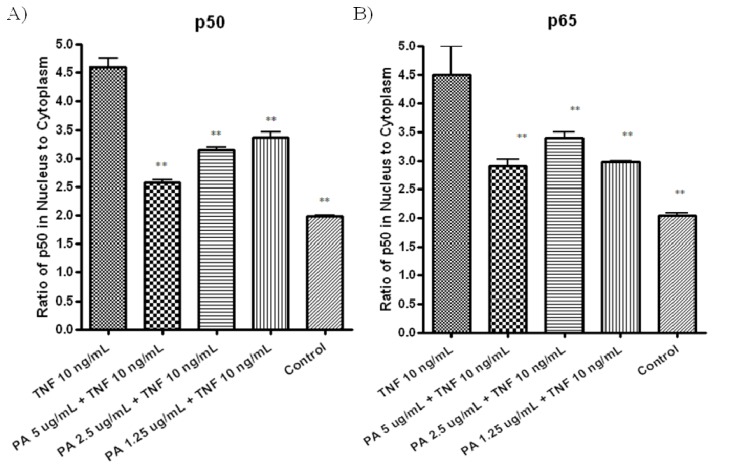
A549 cells were treated with various concentrations of PA for 30 min, followed by treatment with 10 ng/mL TNF-α for another 30 min. Nuclear and cytoplasmic fractions of the A549 treated cells were extracted, the concentration of the active form of (**A**) NF-κB/p50; and (**B**) NF-κB/p65 in both fractions were measured with ELISA kits. Differences of NF-κB/p50, NF-κB/p65 in the nuclear and cytoplasmic fractions are reported.

To further confirm TNF-α-induced NF-κB activation was inhibited in PA treated A549 cells through the inhibition of NF-κB nuclear translocation, NF-κB/p50 (Figure 4A) and NFkB/ p65 (Figure 4B) in nuclear and cytoplasmic fractions of the treated cells were measured by ELISA. Pretreatment with 5 µg/mL of PA resulted in significant decreased in both NF-κB/p50 (Figure 4A) and NF-κB/p65 (Figure 4B) level in nuclear fractions, compared to cells treated with TNF-α control (Figure 4A,B). These results showed that PA inhibits TNF-α-induced NFkB activation by inhibiting nuclear translocation of NF-κB/p50 and NF-κB/p65 in the treated A549 cells.

**Figure 5:**
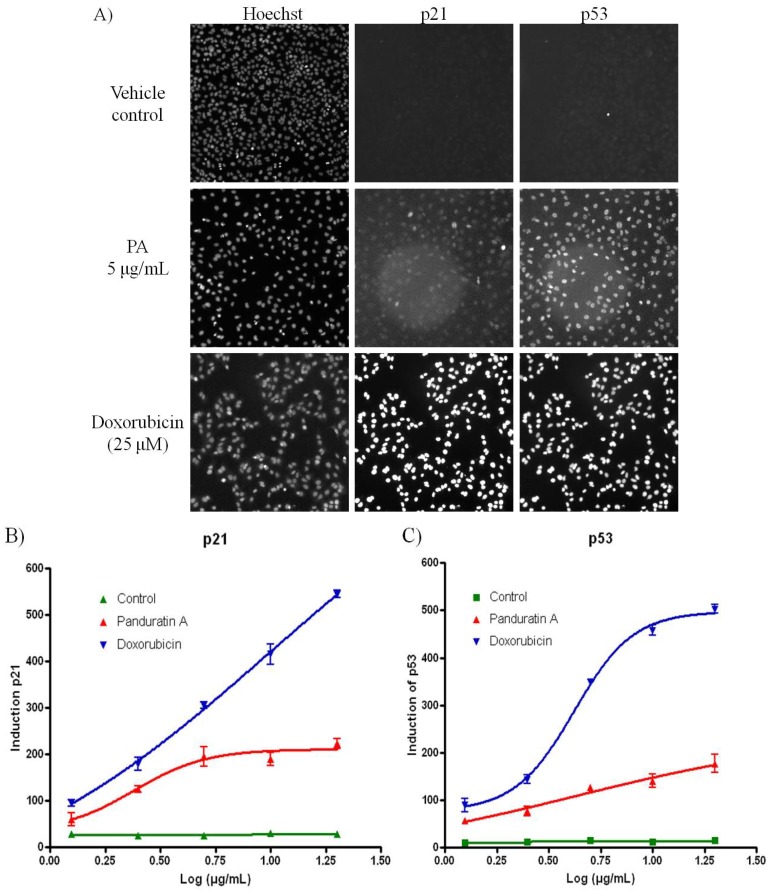
PA induced oxidative stress on A549 cells. (**A**) Fluorescence images acquired on the ArrayScan HCS Reader showing induction of p53 and p21 in the nucleus of A549 cells after 20 h incubation. Doxorubicin was included as positive control. While the untreated cells have minimal p53 or p21 signal, doxorubicin treated cells show bright p53 and p21 labeling in the cell nuclei; (**B**) PA induced p21 was evidenced with increased of the fluorescence signal; (**C**) PA induced p53 was evidenced with increased of the fluorescence signal.

Pretreatment with 5 µg/mL of PA resulted in significant increased in both p53 and p21 level compared to cells treated with vehicle control (Figure 5A,B). The tumour suppressor p53 inhibits cell growth through activation of cell-cycle arrest and apoptosis [8,9]. Besides that, one of the key proteins that modulate the apoptotic response is NF-κB, a transcription factor that can protect or contribute to apoptosis [12,29,30]. Depending on the cell type and the nature of the inducing stimulus, p53 and NF-κB can co-operate each other’s function [31]. Ryazantseva *et al.* [32] also reported that NF-κB is essential for p53-induced cell death. On the other hand, study has been proved that p21 Cip1/Waf1 is activated through NF-ΚB and is an important mediator of this growth arrest response [22,33]. Therefore, we look into the expression level of p53, p21 and 2 of the members of mammalian NF-κB family members which are NF-κB1 (p50) and RelA (p65) [34].

Since previous research had proved that PA inhibited the translocation of NF-κB from cytoplasm to nucleus in PA treated-A549 cells [1], therefore, we further investigated the protein concentration of the active form of NF-κB/p50 and NF-κB/p65 in nuclear fractions. We showed that in PA treated A549 cells; the level of NF-κB/p50 and NF-κB/p65 decreased significantly and increase the expression of p21 and p53. Treatment of A549 cells with PA resulted in a strong inhibition of NF-κB activation, which was consistent with a decrease in nuclear levels of NF-κB/p65 and NF-κB/p50 and the elevation of p53 and p21.

Another study proved that doxycycline treated in osteosarcoma Saos-2 cells showed that NF-κB is essential for p53-induced cell death [32]. The ability of PA in inhibiting NF-κB activation by blocking the nuclear translocation of NF-κB/p65 and NF-κB/p50 transcription factors suggests its promising role in cancer treatment. However, further researches have to prove that the induction of p53 causes an activation of NF-κB that correlates with the ability of p53 to induce apoptosis. 

**Figure 6:**
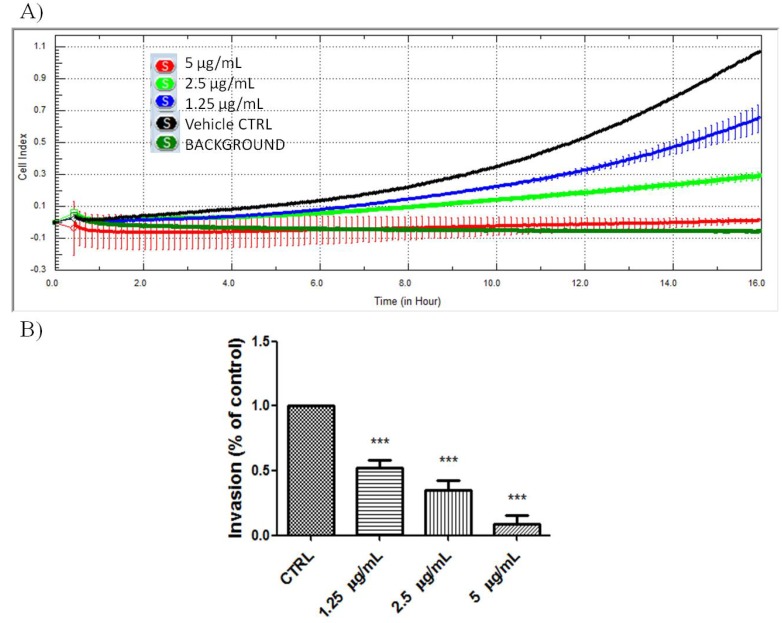
PA inhibited chemoinvasion of A549 cells. (**A**) Representative figure of real-time A549 chemoinvasion profile; (**B**) PA treatment resulted in dose-dependent inhibition of A549 cell chemoinvasion.

NF-κB has been shown to regulate a whole cadre of genes important for angiogenesis, invasion, and metastasis [35,36,37]. Therefore we also look into the invasion inhibition effects mediated by PA in A549 cells. As shown in Figure 6, PA significantly inhibited the invasion of A549 cells in a dose-dependent manner. The chemoinvasion inhibition was observed, even at non-cytotoxic doses.

**Figure 7:**
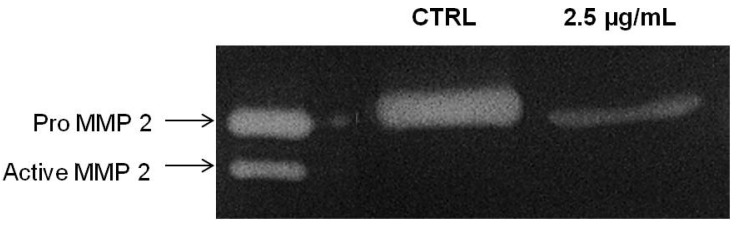
PA inhibited secretion of MMP-2 by A549 cells. Conditioned media of A549 cells treated with PA (2.5 µg/mL) for 24 h and vehicle control were subjected to gelatin zymography.

MMP-2 is important mediator for cancer invasion and metastasis and required in degrading the extracellular matrix and basal membrane degradation during the course of invasion in metastasized cancer [27]. We next tested the correlation of the inhibitory effects of PA on chemoinvasion of A549 cells through Matrigel with the inhibition of MMP-2 secretion. Thus, the conditioned medium of PA treated A549 cells were collected and concentrated for gelatin zymography assay. As showed in Figure 7, PA significantly suppressed the secretion of MMP-2 of A549 cells. Here, we showed that PA suppressed the secretion of MMP-2 and possibly attenuated its activation. The absence of active MMP-2 (62 kDA) moiety in PA-treated samples at 24 h could be perhaps due to physical interference of PA with the intermediate form of MMP-2 which impede its activation through an auto-proteolysis mechanism, or rather, this could be merely due to the decrease in intermediate forms, resulting in the activation of a minute amount of MMP-2 which could not be detected in the experimental condition. 

MMPs are a family of zinc-dependent endopeptidases capable of degrading components of basement membrane and ECM, allowing endothelial cells invading and migrating towards pro-angiogenic factors [38]. In intact cells, MMP-2 is secreted as inactive zymogen (pro MMP-2; 72 kDA), which will be further activated in extracellular milieu by the membrane-type MMP (MT1-MMP) with the aid of TIMP-2 to a 64 kDA active intermediate form, [39] and a subsequent intermolecular autolytic cleavage leads to auto-activation to the 62 kDA activated MMP-2 [40]. Intriguingly, MMP-2 is implicated in promoting cancer cell extravasation, and thus increasing the metastatic potential of tumour [41]. Hence, it is anticipated that inhibitory effects of PA on MMP-2 secretion holds great pharmaceutical value for metastatic malignancy. 

## 3. Experimental

### 3.1. Materials

All solvents (HPLC grade) were purchased from Fisher Scientific (Loughborough, Leicestershire, UK). A549 non-small cell lung cancer cell line was purchased from ATCC (Rockville, MD, USA). RPMI medium, penicillin, streptomycin solution and 0.25% trypsin solution were purchased from Invitrogen (Rockville, MD, USA). MTT, DMSO and heat-inactivated fetal bovine serum and paclitaxel were purchased from Sigma-Aldrich Chemicals (Saint Louis, MO, USA). Cell culture treated 96-well plates and cell culture flasks were purchased from Orange Scientific (Braine-l’Alleud, Belgium).

### 3.2. Plant Materials and Extraction

A voucher specimen of *B. rotunda* with assession No. KU0098 is kept in Phytochemistry Herbarium, University of Malaya. Dried plant materials (4 g) were extracted twice with methanol (50 mL) for 48 h at room temperature, replacing approximately the same volume of fresh methanol after the first 24 h. The extracts were filtered through polyvinypyrrolidone to remove tannins before combining and dried *in vacuo* to obtain the crude extract (0.4 g).

### 3.3. Fractionation and Preparation of Compounds

Isolation of Panduratin A was followed accordingly from Cheah *et al.* 2011 [1]. Panduratin A was subsequently isolated from crude extract by preparative reversed-phase HPLC (Waters Nova-Pak C18 column, particle size 6 μm, 25 × 100 mm) using acetonitrile and water. A gradient of 60% to 100% (v/v) acetonitrile in water at a flow rate of 12 mL/min was applied over 50 min. Identity and purity (>98%) of the isolated panduratin A were determined by analytical HPLC and nuclear magnetic resonance (^1^H-NMR) spectroscopy. The ^1^H-NMR in CDCl_3_ was found to be identical to that previously reported [1,42].

### 3.4. Cells Culture

A549 cell line used in this study was maintained at 37 °C incubator with 5% CO_2_ saturation. Cells were cultured in RPMI media containing 10% FBS and 1% penicillin and streptomycin.

### 3.5. Caspase Assay

Activities of caspase-3 and -8 were measured using the fluorometric assay kit (Calbiochem, Billerica, MA, USA) according to the manufacturer’s instructions. Briefly, cells were treated with PA (5 µg/mL) with or without inhibitors (caspase-3-like: DEVD-CHO; caspase-8: z-IETD-FMK). After treatment, the cells were harvested by trypsinization and cell lysates were prepared as described [43]. Bradford assay was used to measure the protein levels of active caspases in PA-treated cells. The cell lysates were then mixed with reaction buffer and 10 μL of fluorogenic peptide substrate: Ac-DEVD-AMC (caspase-3) and Ac-IETD-AMC (caspase-8), and incubated for 2 h at 37 °C in the dark. The inhibitors were added 30 min before addition of fluorogenic substrate. Wells containing 50 μL of sample buffer, 50 μL of assay buffer and 10 μL of substrate were used as blank. Purified caspase was used as positive control while untreated cell extract was used as negative control. Fluorescent intensity was then measured at excitation of 390 nm and emission of 500 nm. The samples absorbance readings were calculated by subtracting the absorbance of the blank samples. Fold-increase in the protease activity was determined by comparing the levels of the treated cells with untreated controls.

### 3.6. Detection of Cleaved PARP

Cleavage of PARP was detected by immunostaining approach. Briefly, A549 cells treated with PA for 24 h were fixed, permeabilized, blocked and stained with anti-cleaved PARP antibody and secondary antibody conjugated to Dylight 488 Fluorophore. Cisplatin was included as positive control. The nuclei intensity of cleaved PARP was evaluated and quantified on the ArrayScan HCS Reader. 

### 3.7. Oxidative Stress Determination

A549 cells at 80% confluency were treated with indicated concentration of PA for 23.5 h. Then, 50 µL of pre-warmed staining solution containing dihydroethidium (DHE) and Hoechst dye were added into the well, and incubated at 37 °C in 5% CO_2_ for 30 min. The cells were then fixed and the nuclei intensity of ethidium, a product of DHE oxidation was evaluated on the ArrayScan HCS reader.

### 3.8. NF-κB/p50 and NF-κB/p50 p65 Transcription Factor Assay

A549 cells at 70–80% confluence were treated with PA for 30 min, followed by treatment with 10 ng/mL TNF-α for another 30 min. The cells were then washed with PBS and both the nuclear and cytoplasmic fractions of the treated cells were extracted using nuclear extraction kit (Cayman Chemical, Ann Arbour, MI, USA). The concentrations of the active forms of NF-κB/p50 and NF-κB/p65 in both fractions were measured using Cayman NF-κB/p50 and NF-κB/p65 ELISA kits according to the instructions of the manufacturer’s instructions. The differences in NF-κB/p50 and NF-κB/p65 levels between the nuclear and cytoplasmic fractions were reported.

### 3.9. p53 and p21 High Content Screening

The Multiplexed p53 and p21 HITKIT^®^ assays from Cellomics (Pittsburgh, PA, USA) were performed. Briefly, p53 was detected using rabbit anti-p53 primary antibody and DyLight™ 549 conjugated goat anti-rabbit secondary. p21 was detected using mouse anti-p21 primary antibody and Alexa Fluor^®^ 488 conjugated goat anti-mouse secondary antibody. Cell nuclei were labeled with Hoechst 33342.

### 3.10. Image Acquisition and Cytometric Analysis

Plates with stained cells were analyzed using the ArrayScan HCS system (Cellomics). This system is a computerized automated fluorescence imaging microscope that automatically identifies stained cells and reports the intensity and distribution of fluorescence in individual cells. The Array-Scan HCS system scans multiple fields in individual wells to acquire and analyze images of single cells according to defined algorithms. In each well, 1,000 cells were analyzed. Automatic focusing was performed in the nuclear channel to ensure focusing regardless of staining intensities in the other channels. Images were acquired for each fluorescence channel, using suitable filters. Images and data regarding intensity and texture of the fluorescence within each cell, as well as the average fluorescence of the cell population within the well were stored in a Microsoft SQL database for easy retrieval. Data were captured, extracted and analyzed with ArrayScan II Data Acquisition and Data Viewer version 3.0 (Cellomics).

### 3.11. Chemoinvasion Assay

The effects of PA on the chemoinvasion of A549 cells was examine using the RTCA xCELLigence system and CIM-plate 16 (Roche, Mannheim, Germany). The assay was performed according to the manufacturer’s protocol, with minor modifications. The PET membranes of CIM-plate 16 were pre-coated with 0.5 mg/mL of Matrigel. Briefly, serum starved A549 were harvested and seeded onto the upper chamber of a CIM-plate. Complete DMEM supplemented with 10% FBS was placed in the lower chamber to act as a chemoattractant. A control with serum free medium in the lower chamber was included to monitor the background motility of the cells. The effects of PA on the chemoinvasion of A549 cells through Matrigel were monitored in real-time mode for 16 h. 

### 3.12. Gelatin Zymography

The effects of PA on MMP-2 secretion were examined by gelatin zymography. A549 cells in 80% confluency were washed and incubated with fresh serum free medium containing PA for 24 h. The conditioned media were then clarified and concentrated. Equal amount of proteins from treated and untreated control were subjected to gelatin zymography (0.1% gelatin; 10% SDS-PAGE) under non-reducing conditions. After electrophoresis, the gels were washed with renaturing buffer and incubated for 20 h at 37 °C in developing buffer. The gels were subsequently stained with coomasie blue staining solution until clear bands against a blue background were observed. The clear bands represented areas of gelatinolytic activities. Commercially available MMP standards (Calbiochem, Billerica, MA, USA) and molecular marker (Invitrogen, Carlsbad, CA, USA) were separated concurrently for MMP identification. Gel images were acquired on the Bio Rad Chemi XR Gel doc System and the relative intensity of bands was analysed by Quantity One software (Bio-Rad, Hercules, CA, USA).

### 3.13. Statistical Analysis

Each experiment was performed at least two times. Results are expressed as the means value ± standard deviation (SD). Statistical analysis was performed with one-way analysis of variance (ANOVA), with Dunnett's Multiple Comparison Test to identify between-group differences using GraphPad Prism software (version 4.0; GraphPad Software Inc., San Diego, CA, USA). Statistical significance is expressed as *** *p* < 0.001; ** *p* < 0.01; * *p* < 0.05. Log IC_50_ calculations were performed using the built-in algorithms for dose-response curves with variable slope. A fixed maximum value of the dose-response curve was set to the maximum obtained value for each drug.

## 4. Conclusions

Treatment of A549 cells with PA resulted in a strong inhibition of NF-κB activation, which was consistent with a decrease in nuclear levels of NF-κB/p65 and NF-κB/p50 and the elevation of p53 and p21. The ability of PA in inhibiting NF-κB activation by blocking the nuclear translocation of NF-κB/p65 and NF-κB/p50 transcription factors suggests its promising role in cancer treatment. Our findings not only prove the effects of PA, but may also be important in the design of therapeutic protocols that involve targeting of either p53 or NF-κB. In summary, our results strongly indicates the potential of PA as an potential anti-cancer agent, with its multipotential effects in inhibiting survival and proliferation of A549 cells, chemoinvasion, and secretion and activation of MMP-2. Further studies should be carried out to investigate the mechanism of inhibitory action, such as quantitative analysis of IκB kinase activity, IκB alpha phosphorylation and degradation, p50 and p65 nuclear translocation, DNA binding and NF-κB-dependent reporter gene expression.
